# Supplementary material for: Parabrachial Ntsr1 neurons modulate food intake and anxiety through a projection to the ventromedial hypothalamus
Source: Proc Natl Acad Sci U S A. 2026 Jul 22;123(30):e2605466123. doi: 10.1073/pnas.2605466123 (PMC13416325; doi:10.1073/pnas.2605466123)
Supplement: Supplementary file 1 — Appendix 01 (PDF) [file pnas.2605466123.sapp.pdf]

Supporting Information for

Parabrachial *Ntsr1* neurons modulate food intake and anxiety through a projection to the ventromedial hypothalamus

Jordan L. Pauli[1,2], Sekun Park[1,2], Rachel R. Felix[1], and Richard D. Palmiter[1,3]

1. Howard Hughes Medical Institute and Department of Biochemistry, University of Washington, Seattle WA 98195
2. Co-first authors
3. Contact information: palmiter@uw.edu

Richard Palmiter
Email: palmiter@uw.edu

This PDF file includes:

Figures S1 to S10

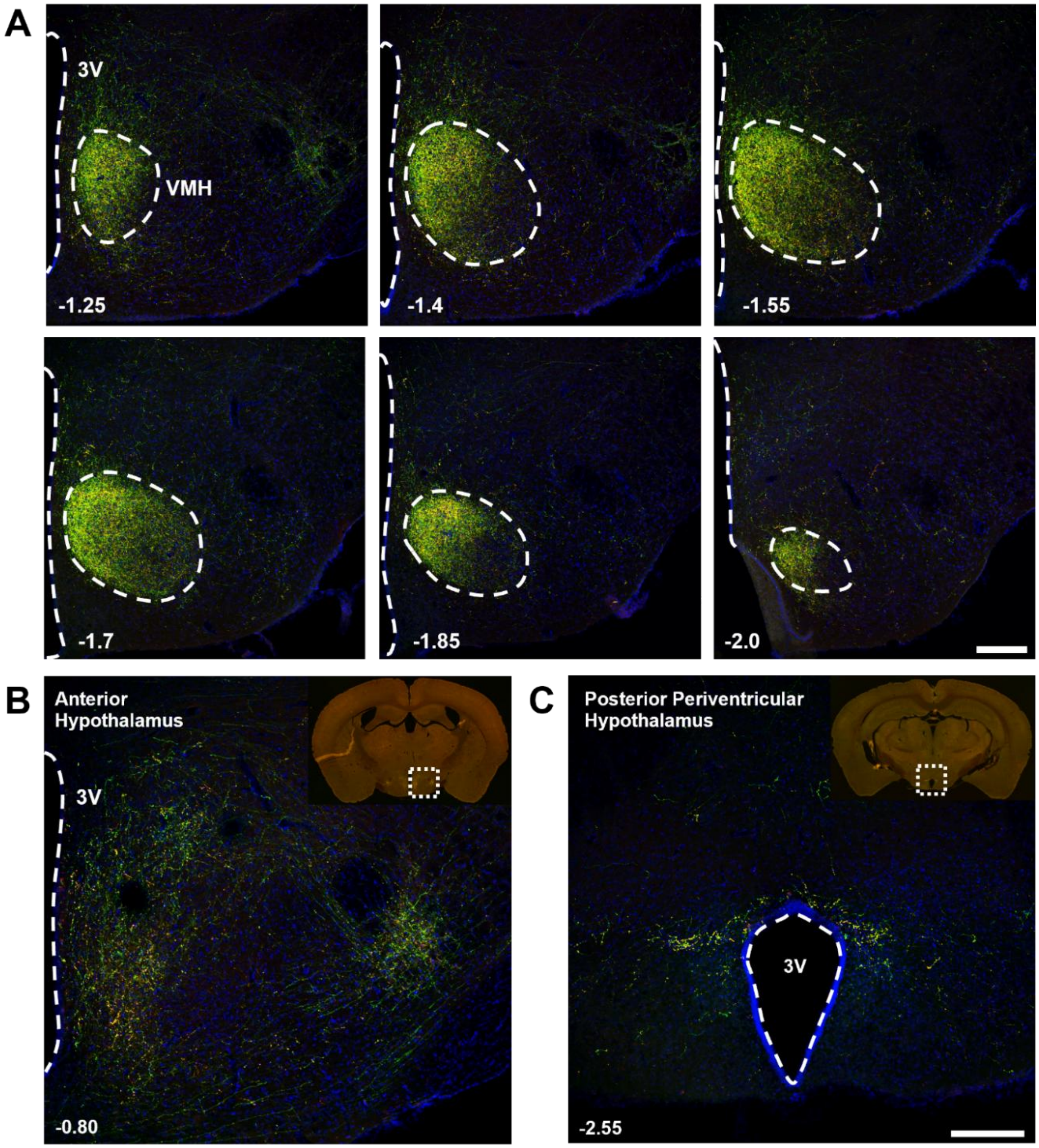

Fig. S1. *Ntsr1*^{PBN} neuronal projections in the hypothalamic regions.
(A) Images showing the pattern of PBN *Ntsr1* innervation throughout the VMH.
(B) Images of PBN *Ntsr1* innervation in the anterior hypothalamus and C the posterior periventricular hypothalamus. (pictures in A-C were taken at the same settings; the average synaptophysin (red) pixel intensity in (B) and (C) was 19.84% and 19.80%, respectively, compared to that in (A). (*n* = 8 hypothalamus sections from 2 mice)
Scale bars: 200 µm. 3V, third ventricle; VMH, ventromedial hypothalamus
Numbers in lower left indicate bregma level.

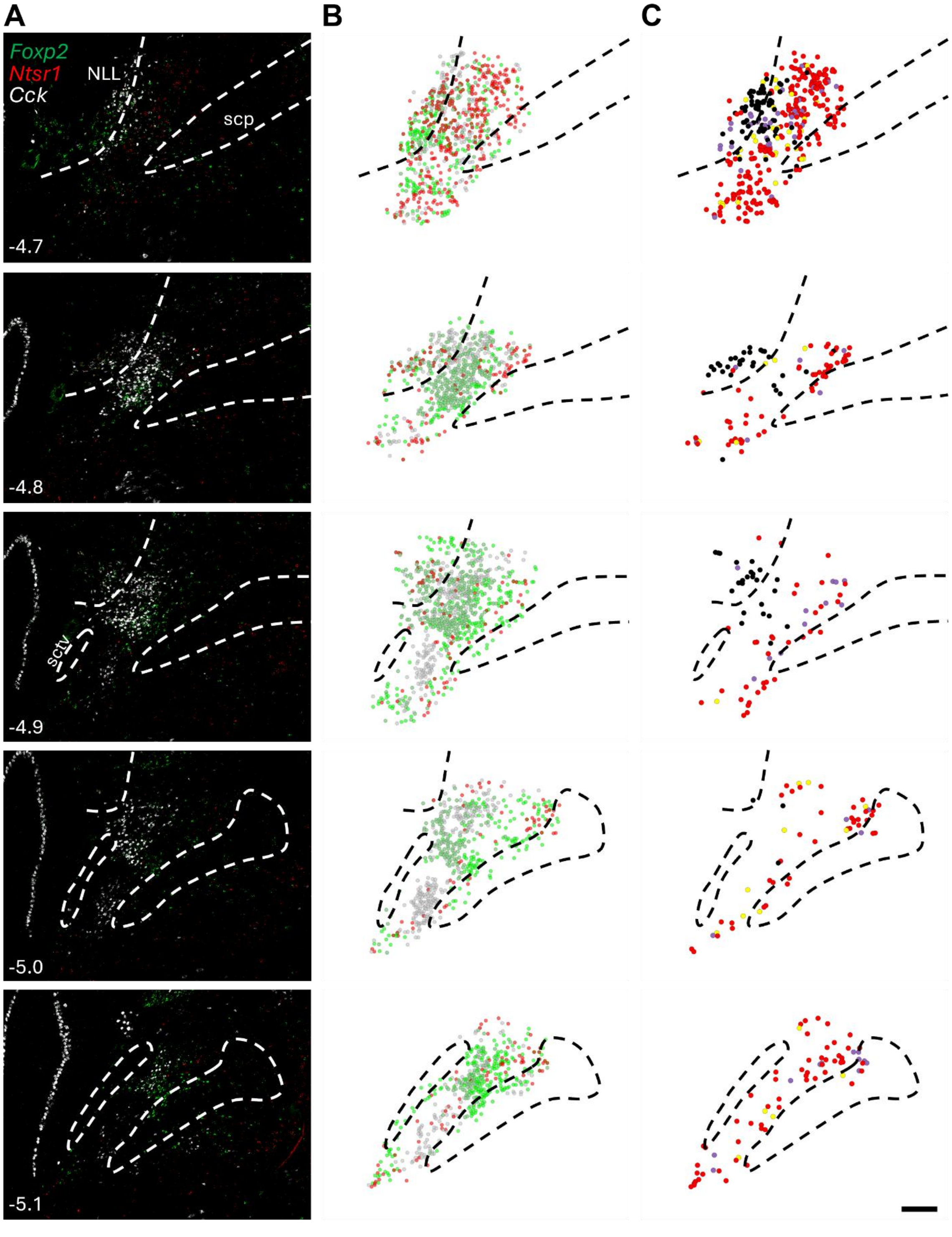
A
B
C
Foxp2
Ntsr1
Cck
NLL
scp
sctv
-4.7
-4.8
-4.9
-5.0
-5.1

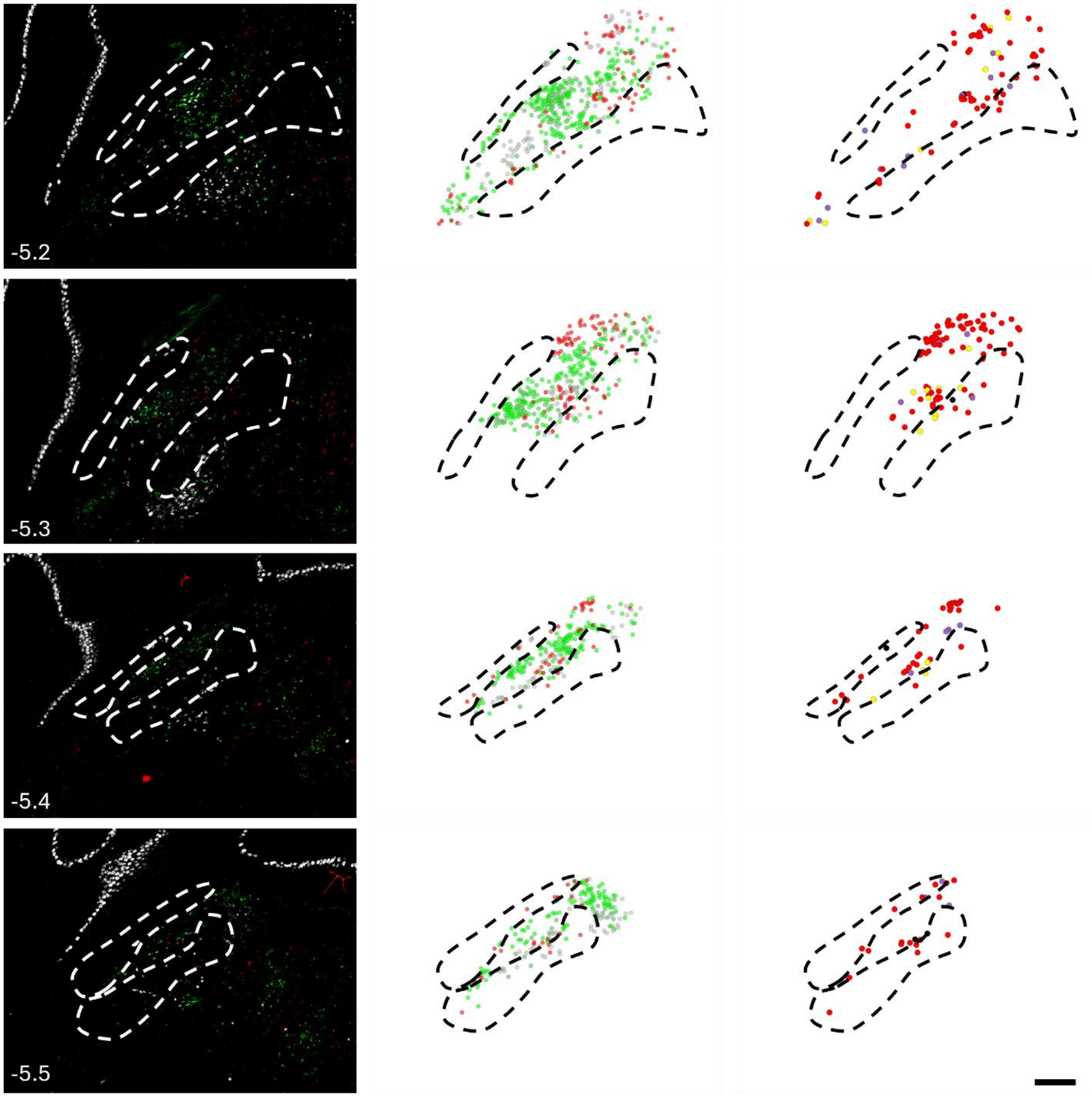

Fig. S2. Spatial characterization of PBN *Ntsr1*, *Cck*, and *Foxp2* throughout PBN.
(A) Representative images of the left PBN of one animal showing fluorescent *in situ* staining of *Foxp2*, *Ntsr1*, and *Cck*.
(B) Dot map of the same PBN sections showing *Foxp2* (green), *Ntsr1* (red), and *Cck* (grey) probes location based on the lateral PBN only.
(C) Dot map of PBN *Ntsr1* (red), *Ntsr1* and *Cck* (yellow), *Ntsr1* and *Foxp2* (Purple) and triple labeled (black) cells.
Scale bars: 200 µm. Numbers in lower left indicate bregma level.

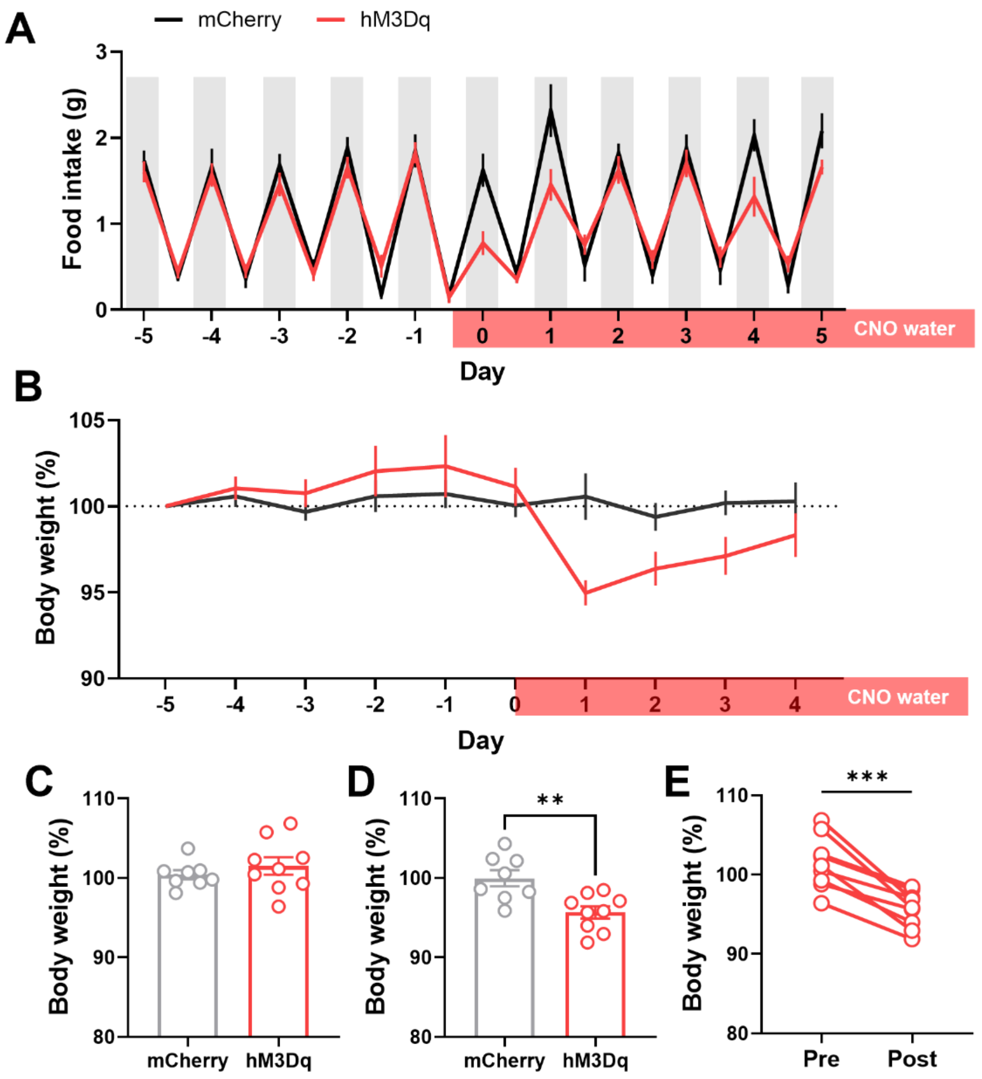

Fig. S3. Patterns of food intake over the entire chronic feeding timeline.
(A) Graph showing food intake over 5 days before and after CNO water in mCherry and hM3Dq mice. Red bar indicates CNO water periods and grey bars show dark cycles (*n* = 8 mice for mCherry and *n* = 8 mice for hM3Dq).
(B-E) Comparison of body weight changes based on baseline (day -5) over the chronic CNO exposure feeding experiment before and after introduction of CNO water. Quantification of body weight (C) before CNO water, (D) after CNO water and (E) individual animals in hM3Dq group (*n* = 8 mice for mCherry and *n* = 9 mice for hM3Dq, unpaired two-tailed Student's t-test, $p = 0.4092$ for (C); $p = 0.0036$ for (D); paired two-tailed t-test, $p = 0.0002$ for (E).
Data are presented as mean ± SEM.

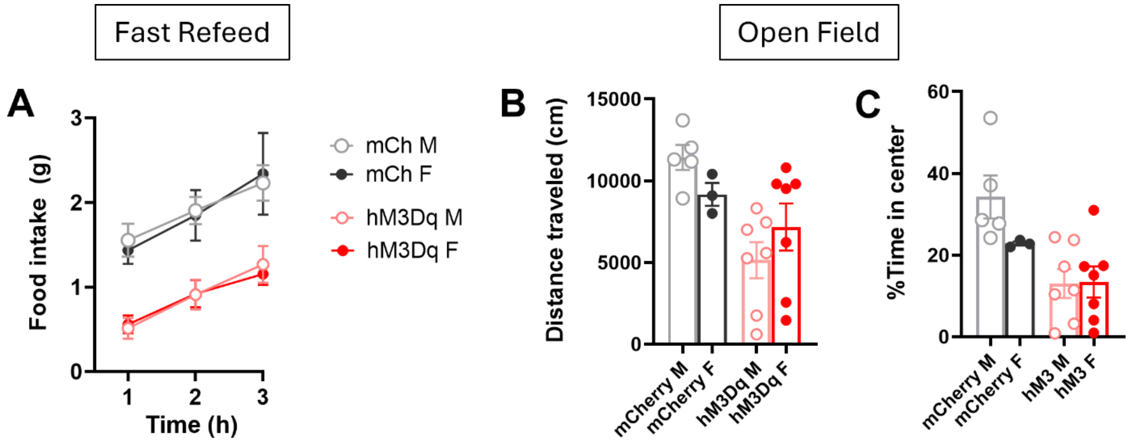

Fig. S4. Feeding and movement are the same in both sexes.
(A) Comparison in food intake after an overnight fast between female and male mCherry and hM3Dq mice (*n* = 5 and 3 for male and female in mCherry group, Two-way RM ANOVA, *p* = 0.9473; *n* = 7 for both male and female in hM3Dq group, Two-way RM ANOVA, *p* = 0.9299).
(B) Comparison in distance traveled in the open field between female and male mCherry and hM3Dq mice (*n* = 5 and 3 for male and female in mCherry group, *p* = 0.0959; *n* = 7 for both male and female in hM3Dq group, unpaired two-tailed Student's t test; *p* = 0.2865).
(C) Comparison in percentage of time spent in the center of the open field between female and male mCherry and hM3Dq mice (*n* = 5 and 3 for male and female in mCherry group, *p* = 0.1550; *n* = 7 for both male and female in hM3Dq group, unpaired two-tailed Student's t test; *p* = 0.9428).
Data are presented as mean ± SEM.

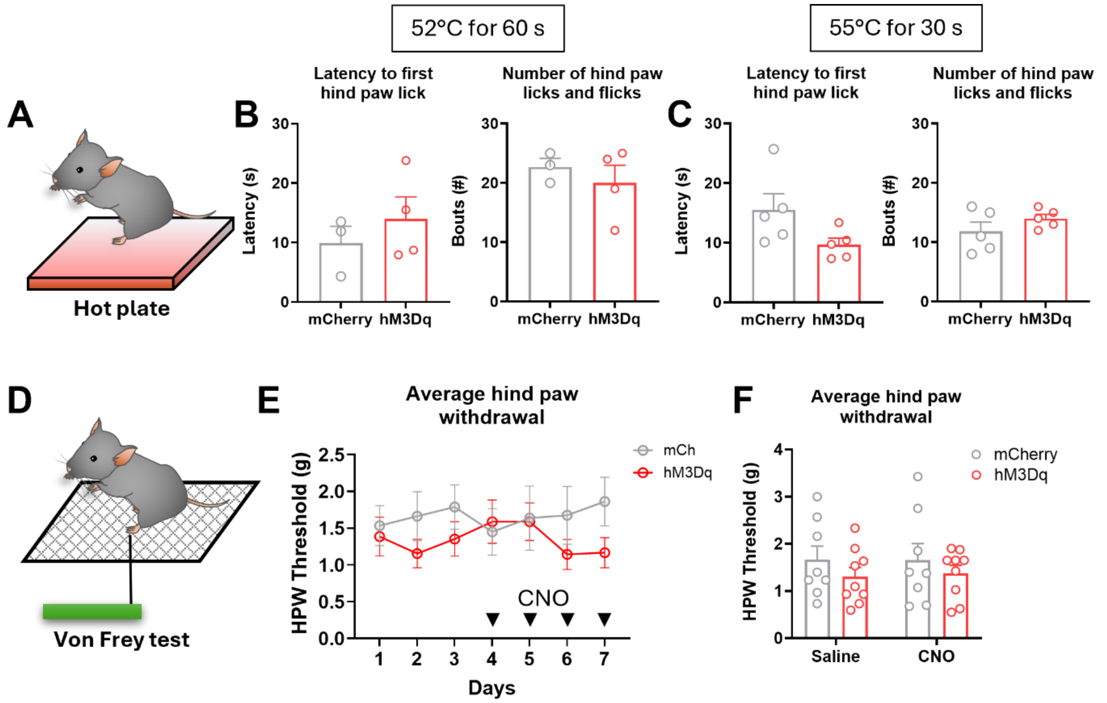

Fig. S5. PBN *Ntsr1* neuronal activation has no effect on heat or mechanical sensitivity.
(A) Illustration showing hot plate test.
(B) Comparisons in latency to first hind paw lick and number of hind paw licks and flicks in a 52 °C hot plate (n = 3 for mCherry mice and n = 4 for hM3Dq mice, unpaired two-tailed Student's t-test; p = 0.4486 and p = 0.5056).
(C) Comparisons in latency to first hind paw lick and number of hind paw licks and flicks in a 55°C hot plate (n = 5 for mCherry mice and n = 5 for hM3Dq mice, unpaired two-tailed Student's t-test; p = 0.0864 and p = 0.2426).
(D) Illustration showing the schematic showing von Frey filament application to the hindpaw.
(E) Paw withdrawal threshold for 7 days. CNO was given on days 4-7 and is marked by arrows (n = 8 mCherry for and n = 9 for hM3Dq mice Two-way RM ANOVA p = 0.3394 for mCherry vs hM3Dq).
(F) Bar graphs showing the total averaged hind paw withdrawal thresholds during saline and CNO applications (n = 8 for mCherry and n = 9 for hM3Dq mice (Two-way RM ANOVA p = 0.5340 for Saline, p = 0.6782 for CNO).
Data are presented as mean ± SEM.

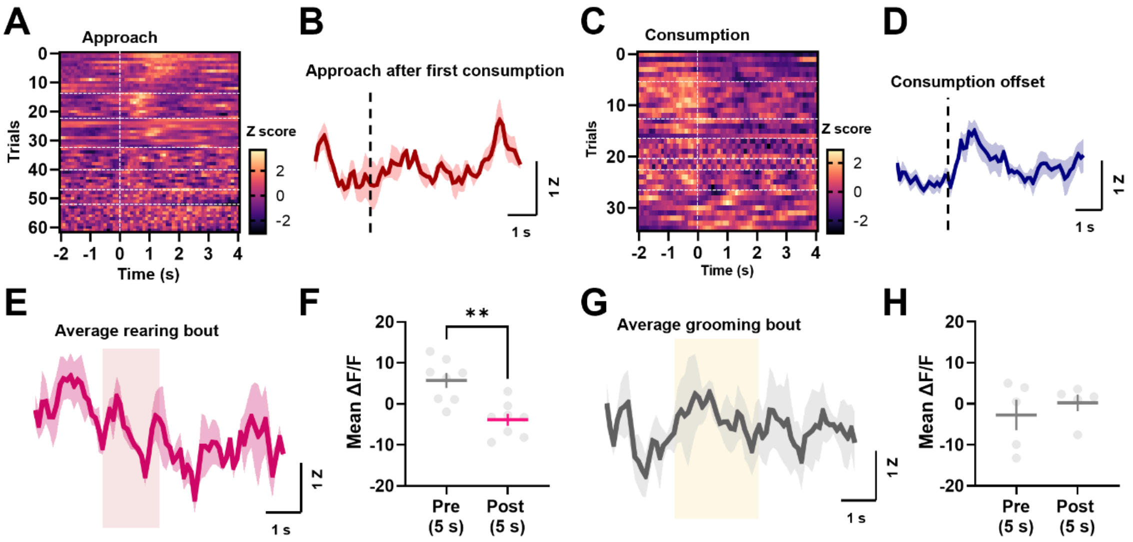

Fig. S6. PBN *Ntsr1* neuronal activities during food intake.
(A) Heat map of neural activity across approach behaviors from all mice. White dotted horizontal lines indicate individual mice (n = 61 trials in 7 mice).
(B) Average trace of neural activities during approach behaviors after the first consumption (n = 5 mice).
(C) Heat map of neural activity across consumption behaviors from all mice. White dotted horizontal lines indicate individual mice (n = 34 trials in 7 mice).
(D) Average trace of neural activities after consumption behaviors aligned by consumption offset (n = 5 mice).
(E) Average trace of rearing behaviors during food intake experiment (n = 3 mice).
(F) Mean fluorescence during the pre 5 s and post 5 s of rearing onset (n = 8 bouts in 3 mice, paired two-tailed Student's t-test; p = 0.0040).
(G) Average trace of grooming behaviors during food intake experiment (n = 3 mice).
(H) Mean fluorescence during the pre 5 s and post 5 s of grooming onset (n = 5 bouts in 3 mice, paired two-tailed Student's t-test; p = 0.5527).
Data are presented as mean ± SEM.

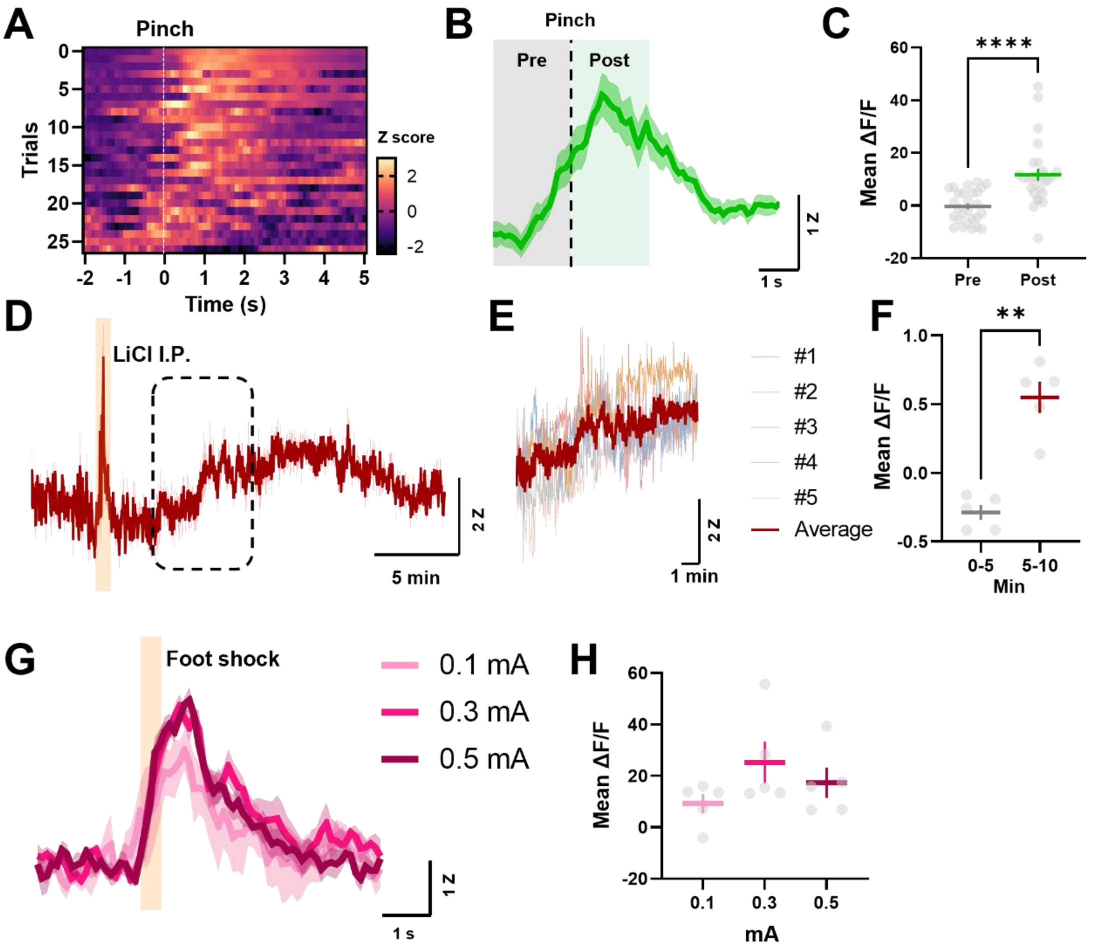

Fig. S7. PBN *Ntsr1* neuronal activities in response to aversive stimuli.
(A) Heat map of neural activity in response to tail pinch from all mice. White dotted horizontal lines indicate individual mice (n = 26 trials in 7 mice).
(B) Average trace of neural activities during tail pinch (n = 7 mice).
(C) Mean fluorescence during the pre 2 s and post 2 s of tail pinch (n = 26 trials in 7 mice, paired two-tailed Student's t-test; $p < 0.0001$).
(D) Average trace of neural activities in response to LiCl administration. Yellow box indicates approximate time of IP injection (n = 5 mice).
(E) Magnified view of individual and average traces corresponding to the dotted box in (D).
(F) Mean fluorescence during 0-5 min and 5-10 min after LiCl administration (n = 5 mice, paired two-tailed Student's t-test; p = 0.0021).
(G) Average neural activities in response to various intensity of electric foot shock. Yellow box indicates shock duration (n = 5 mice).
(H) Mean fluorescence in response to different shock intensities (n = 5 mice, One-way RM ANOVA).
Data are presented as mean ± SEM.

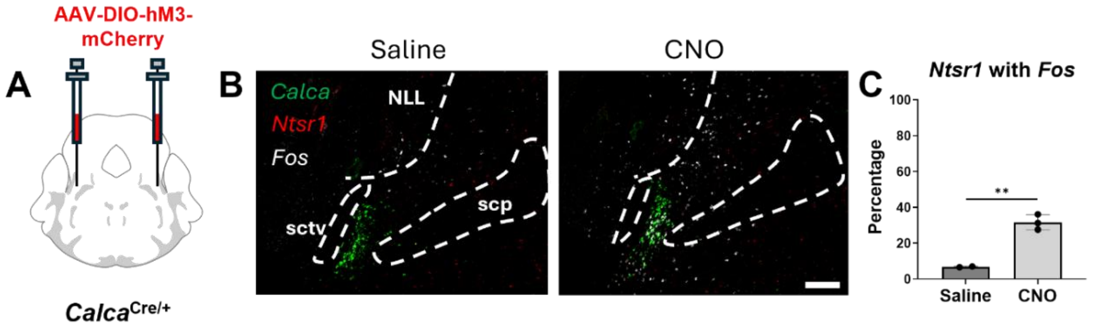

Fig. S8. PBN *Calca* neuronal activation results in *Fos* expression in PBN *Ntsr1* neurons.
(A) Schematic of viral injection of AAV_{DJ}-DIO-hM3Dq-mCherry into the PBN of *Calca*$^{Cre/+}$ mice
(B) Representative image of expression pattern of *Calca*, *Ntsr1*, and *Fos* in the PBN Saline or CNO administration
(C) Activation of PBN *Calca* neurons with CNO increased *Fos* mRNA in PBN *Ntsr1* neurons (*n* = 2 PBN sections for saline, *n* = 3 PBN sections for CNO, unpaired two-tailed Student's t test; p<0.0045)
Scale bar: 200 µm. Data are presented as mean ± SEM.

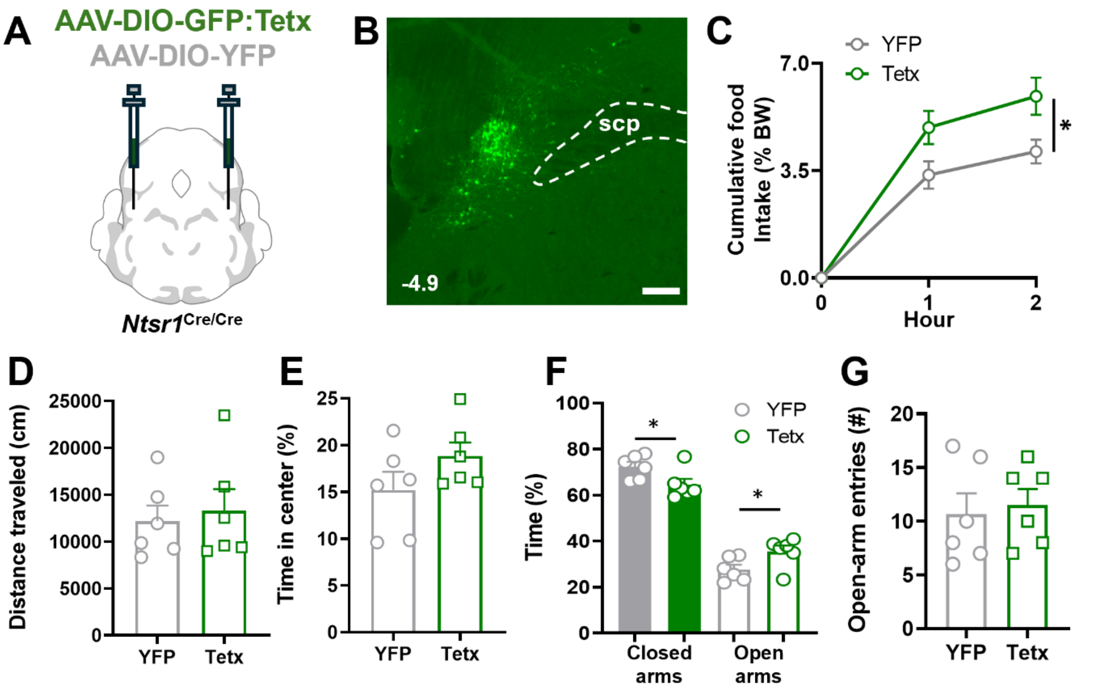

Fig S9. Silencing *Ntsr1* neurons increases food intake and reduces anxiety.
(A) Schematic of viral injection of AAV-DIO-GFP:Tetx into the PBN of *Ntsr1*$^{Cre/Cre}$ mice.
(B) Representative image of Tetx expression in PBN.
(C) Cumulative food intake calculated as percent body weight (Two-way RM ANOVA; p = 0.0341 for YFP vs Tetx).
(D) Distance traveled in the open field test (Unpaired two-tailed Student's t-test; p = 0.6999).
(E) Percent time in center during open field (Unpaired two-tailed Student's t-test; p = 0.1645).
(F) Percent time spent in closed and open arms in the elevated plus maze (Student t-test; p = 0.0386 for closed arms and p = 0.0385 for open arms).
(G) Total number of open arm entries in the elevated plus maze (Unpaired two-tailed Student's t-test; p = 0.7399).
Scale bars, 200 µm, n = 6 for YFP and n = 6 for Tetx mice. Data are presented as mean ± SEM.

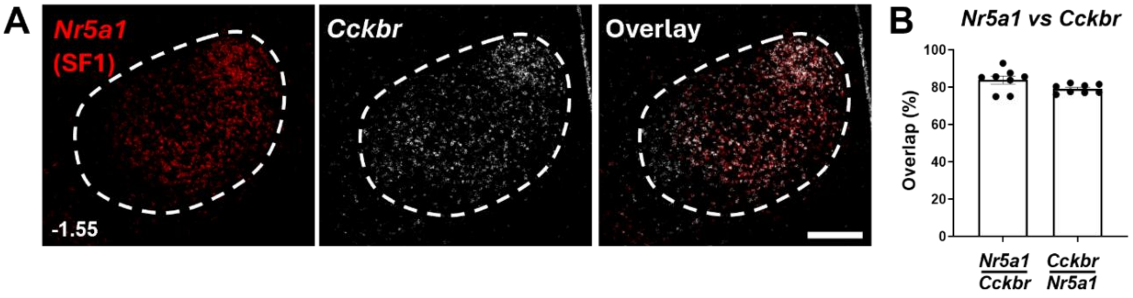

Fig. S10. *Nr5a1* neurons in the VMH coexpress *Cckbr.*
(A) Representative images of *in situ* hybridization for *Nr5a1* (red), *Cckbr* (white).
(B) Percent overlap between *Cckbr* and *Nr5a1* positive populations in the VMH (n = 8 whole VMH for both groups).
Scale bars: 200 µm. Numbers in lower left indicate bregma level. Data are presented as mean ± SEM.
